# Genome-wide association analysis identifies candidate loci associated with hydroxycinnamic acid accumulation in sweetpotato

**DOI:** 10.3389/fpls.2026.2001245

**Published:** 2026-10-15

**Authors:** Yiren Su, Lingxiao Zhao, Shizhuo Xiao, Xibin Dai, Zhilin Zhou, Qinghe Cao

**Affiliations:** 1 School of Life Sciences and Food Engineering, Jiangsu Normal University, Xuzhou, China; 2 Key Laboratory of Sweetpotato Biology and Genetic Breeding, Ministry of Agriculture and Rural Affairs, Xuzhou Institute of Agricultural Sciences in Jiangsu Xuhuai District, Xuzhou, China

**Keywords:** abiotic stresses, candidate genes, GWAS, hydroxycinnamic acid-related compounds, sweetpotato

## Abstract

Hydroxycinnamic acid-related compounds are important phenolic bioactive constituents in sweetpotato storage roots and play important roles in quality formation and abiotic stress responses. In this study, the contents of 33 hydroxycinnamic acid-related compounds were determined in 229 sweetpotato germplasm accessions grown in three agroecological regions. Genome-wide association analysis (GWAS) was conducted to identify candidate genes, and the relationships of these genes with hydroxycinnamic acid-related compounds accumulation and abiotic stress responses were further investigated. The results showed that the 33 hydroxycinnamic acid-related compounds exhibited extensive natural variation within the population. Comprehensive evaluation identified several high-accumulation accessions, including BZ13, BZ31, BZ19, BZ20, and BZ30. GWAS identified 4,938 significant SNP–trait associations. Stable signals for FA-4-O-GlcA and isoFer-C1-GlcA were detected in the 19.71–21.09 Mb interval on Iba_chr05a, whereas recurrent signals on Iba_chr10a were concentrated in the 24.88–25.10 Mb and 25.54–26.14 Mb intervals. Candidate gene analysis identified genes encoding caffeoylshikimate esterase (CSE), UDP-glycosyltransferases (UGTs), ATP-binding cassette transporters (ABC transporters), and several transcription factors. Transcriptome analysis under stress conditions further showed that several candidate genes were differentially expressed under drought, heat, and cold stresses, indicating that they may also participate in abiotic stress responses in sweetpotato. Overall, this study revealed the natural variation and genetic basis of hydroxycinnamic acid-related compounds in sweetpotato and identified candidate loci and genes associated with both hydroxycinnamic acid-related compounds accumulation and abiotic stress responses, providing candidate loci for subsequent molecular marker development and useful genetic resources for functional quality improvement and stress-tolerant breeding in sweetpotato.

## 1 Introduction

Sweetpotato (*Ipomoea batatas* (L.) Lam.) is one of the world’s most important root crops (Yan et al., 2024). Owing to its strong adaptability (Ojwang et al., 2023), high yield (Lee et al., 2021), and tolerance to nutrient-poor soils (Grüneberg et al., 2022), sweetpotato plays an important role in food security and nutritional provision in developing countries (Xiao et al., 2021). In addition to essential nutrients such as starch, dietary fiber, and vitamins (Katayama et al., 2017), sweetpotato is rich in various bioactive compounds (Wei et al., 2025), among which phenolic acids constitute an important class (Luo et al., 2013). Previous studies have demonstrated that phenolic acids contribute to the formation of plant flavor (Qi et al., 2024 and Su et al., 2026), coloration (Qian et al., 2023), and processing quality (Najman et al., 2023). For example, in tea plants (*Camellia sinensis* (*L.*) Kuntze), phenolic acid accumulation is closely associated with the bitterness and astringency of tea infusions (Xu et al., 2026), whereas in tobacco (*Nicotiana tabacum L*.), phenolic acid accumulation influences aroma quality and taste characteristics (Li et al., 2025a). Furthermore, phenolic acids play important roles in plant growth and development (Xie et al., 2023; Yadav et al., 2020) and contribute to plant responses and adaptation to abiotic stresses (Liu et al., 2026), including drought (Ren et al., 2026) and heat stress (Sharma et al., 2019).

Based on their chemical structures, phenolic acids can be classified into hydroxybenzoic acids and hydroxycinnamic acids (Khawula et al., 2023). Hydroxycinnamic acid-related compounds are a widely distributed class of phenolic acid derivatives in plants and have considerable physiological significance and broad application potential (Sengupta et al., 2019). *In vitro* and *in vivo* studies have demonstrated that hydroxycinnamic acid-related compounds possess various biological activities, including antioxidant (Valiñas et al., 2015), antimicrobial (De Camargo et al., 2017), anti-inflammatory (Spano et al., 2022), and antitumor (Ceylan et al., 2025); consequently, they have been widely used in the pharmaceutical, food, and personal-care industries. hydroxycinnamic acid-related compounds mainly include caffeic acid, ferulic acid, p-coumaric acid, and caffeoylquinic acid derivatives (Hong et al., 2025; Das et al., 2021), represented by chlorogenic acid (Cunha et al., 2024). These compounds are important products of the phenylpropanoid pathway (Song et al., 2026), which is further connected to the biosynthesis of flavonoids, lignin, and various phenolic acid derivatives and is governed by a complex regulatory network involving both structural genes and transcription factors (Zhang et al., 2021). *MdMYB93* regulates *MdHCT6* expression, increasing chlorogenic acid and caffeic acid contents in apple calli by 2.6- and 1.4-fold, respectively (Xie et al., 2025). *LmMYB111* activates the expression of genes such as *LmPAL*, *Lm4CL2*, and *LmCHI*, thereby promoting the accumulation of chlorogenic acid and luteoloside in honeysuckle (Liu et al., 2025). In sweetpotato, chlorogenic acid and caffeoylquinic acid derivatives are the major hydroxycinnamic acid derivatives (Islam et al., 2016; Tanaka et al., 2017). Metabolomic analyses further revealed significant differences in phenolic acid accumulation among sweetpotato storage roots with different flesh colors (Wan et al., 2024). In addition, a study based on natural allelic variation demonstrated that the *SPWR1*–*SPWR2* regulatory module affects the accumulation of quinic acid and its derivatives, suggesting that secondary metabolite accumulation in sweetpotato is genetically regulated (Liu et al., 2022a). Regarding hydroxycinnamic acid biosynthesis, *IbHQT* has been shown to participate in chlorogenic acid formation (Xiang et al., 2026), while overexpression of *IbPAL1* promotes chlorogenic acid accumulation, indicating that key structural genes in the phenylpropanoid pathway contribute to phenolic acid biosynthesis (Yu et al., 2021). More recently, the transcription factor *IbGLK1* was shown to promote chlorogenic acid accumulation by regulating the expression of chlorogenic acid biosynthesis-related genes, including *IbC4H*, *IbHCT*, *IbHQT*, and *IbUGCT* (Luo et al., 2024). However, sweetpotato is a highly heterozygous hexaploid crop (Yan et al., 2022b), and its complex genetic background has hindered the identification of genes involved in hydroxycinnamic acid-related compounds biosynthesis and the elucidation of their molecular regulatory mechanisms.

Genome-wide association study (GWAS) is a gene-mapping approach based on linkage disequilibrium in natural populations (Chen et al., 2025b). Because it does not require the construction of specialized genetic populations, GWAS has become an important tool for dissecting the genetic architecture of complex quantitative traits in crops (Guo et al., 2026). In eggplant (*Solanum melongena* L.), GWAS identified a major-effect locus controlling the accumulation of dicaffeoylquinic acids and revealed that mutations in a *GDSL-like* esterase/lipase gene within this region reduced the contents of chlorogenic acid derivatives in fruits (Gaccione et al., 2025). In apple (*Malus domestica* Borkh.), GWAS identified a *Myb9-like*–*LAR* regulatory module associated with proanthocyanidin accumulation, in which a SNP located 315 bp upstream of *LAR* promoter was significantly associated with variation in the contents of multiple tannin compounds (Lin et al., 2023). In sweetpotato, Xiao et al. (2023) performed whole-genome resequencing and GWAS using 314 germplasm accessions, revealing the population genetic structure of sweetpotato and providing a valuable reference for the genetic improvement of important traits. By integrating GWAS with transcriptome analysis, Liu et al. (2022b) identified five SNP loci significantly associated with storage-root flesh color and predicted that candidate genes such as *IbF3′H* may participate in anthocyanin biosynthesis, thereby influencing flesh color formation. An et al. (2026) found that anthocyanin accumulation in sweetpotato leaves was predominantly regulated by the tandem *IbMYB2*–*IbMYB3* gene cluster on chromosome 5, whereas anthocyanin biosynthesis in storage roots also involved structural genes such as *IbAOMT*, *Ib3GGT*, and *IbLDOX*. These studies have laid a foundation for dissecting the genetic basis of quality traits in sweetpotato. However, multi-environment GWAS of hydroxycinnamic acid-related compounds in sweetpotato storage roots remains limited.

To elucidate the genetic basis underlying hydroxycinnamic acid-related compounds accumulation in sweetpotato storage roots, 229 germplasm accessions were evaluated across three environments, including Xuzhou (XZ), Wenchang (WC), and Jinan (JN). The contents of 33 hydroxycinnamic acid-related compounds derivatives were quantified, and genome-wide association studies (GWAS) were performed using a mixed linear model (MLM). Through multi-environment association analysis, linkage disequilibrium (LD) analysis, genotype effect evaluation, and functional annotation, key candidate genes associated with hydroxycinnamic acid-related compounds accumulation were identified. Their potential biological functions were further assessed by analyzing expression patterns under different genetic backgrounds and under drought, heat, and cold stress conditions. This study provides insights into the genetic architecture and abiotic stress-related responses of hydroxycinnamic acid-related compounds accumulation in sweetpotato and provides valuable resources for molecular breeding of germplasm with high hydroxycinnamic acid contents.

## 2 Materials and methods

### 2.1 Plant materials and field experiments

A total of 229 sweetpotato germplasm accessions originating from China and other countries were used in this study. All accessions were obtained from the National Sweetpotato Germplasm Repository in Xuzhou, Jiangsu Province, China (117.30° E, 34.28° N). Detailed information on the germplasm accessions is provided in **Supplementary Table S1**. In 2020, field experiments were conducted in three agroecological regions: Wenchang, Hainan Province (110.80° E, 19.54° N); Xuzhou, Jiangsu Province (117.31° E, 34.27° N); and Jinan, Shandong Province (116.85° E, 36.35° N). Each sweetpotato accession was evaluated using three biological replicates. Each replicate corresponded to one independent planting row containing 10 plants, resulting in 30 plants per accession. Each plot consisted of three experimental rows and one border row, with an approximate plot size of 2.0 m × 1.4 m (2.8 m²). The plant spacing within rows was 20 cm, and a 0.2 m drainage furrow was maintained between adjacent rows. An 80 cm isolation distance was maintained between adjacent accessions to minimize potential interference among different genotypes.

All accessions were cultivated under the same field management practices at each experimental site. Fertilization and irrigation were performed according to local conventional sweetpotato production practices. At 15–20 days after planting, urea was applied at 5–8 kg mu^-^¹ (approximately 75–120 kg ha^-^¹) only when seedling growth was relatively weak to promote plant establishment. During drought periods, furrow irrigation was applied, and the irrigation water level was maintained below one-third of the ridge height. During the rainy season, drainage channels were kept clear to prevent waterlogging.

### 2.2 Sample preparation

After 120 days of cultivation, storage roots were harvested. Healthy storage roots without disease symptoms, insect damage, or mechanical injury were selected. Storage roots similar in size and developmental status were selected. For each biological replicate, three representative storage roots were randomly selected and pooled as one sample for hydroxycinnamic acid-related compounds metabolite analysis. The three biological replicates were processed and analyzed independently, and samples from different replicates were not pooled. The pooled storage root samples were washed, peeled, and cut into uniformly sized pieces. Six grams of fresh sample was accurately weighed and mixed with 35 mL of acetone–methanol–formic acid extraction solution (25:75:1, v/v/v). The samples were homogenized for 5 s using a Vitamix 5200 homogenizer (Vitamix, Cleveland, OH, USA), and this procedure was repeated three times. The homogenates were then ultrasonically extracted at 200 W for 15 min at room temperature and centrifuged at 4,000 g for 20 min. The supernatant was collected and concentrated to 2 mL using a rotary evaporator (N-1300D-WB; Tokyo Rikakikai Co., Tokyo, Japan) for subsequent LC–MS/MS analysis.

### 2.3 LC–MS/MS analysis

Hydroxycinnamic acid-related compounds were analyzed using an ultra-high-performance liquid chromatography system coupled to a TripleTOF 4600 high-resolution mass spectrometer (AB Sciex, Framingham, MA, USA). Chromatographic separation was performed on a Halo C18 column (150 mm × 2.1 mm, 2.7 μm; Advanced Materials Technology, Wilmington, DE, USA) maintained at 45 °C, with a flow rate of 0.3 mL/min. Mobile phase A consisted of water containing 0.1% formic acid, whereas mobile phase B consisted of a methanol–acetonitrile mixture (1:1, v/v) containing 0.1% formic acid. The gradient elution program was as follows: 0–5 min, 10–60% B; 5–10 min, 60–80% B; 10–15 min, 80–100% B; and 15–20 min, 100% B. Mass spectrometric detection was performed in positive-ion atmospheric-pressure chemical ionization (APCI) mode. Sodium formate solution was used for mass calibration, and the scan range was m/z 100–2,000. Raw data were processed using PeakView software (v2.2; AB Sciex), and peak extraction, alignment, and filtering were performed using the XCMS Online platform. Compounds were identified based on accurate mass and tandem mass spectrometric fragmentation patterns by comparison with a laboratory-built hydroxycinnamic acid-related compounds database developed by our research group and the JSNUPOLYENE v2.7 database. Quantification was performed using an external calibration curve established with a caffeic acid (CA) standard. The calibration equation was Y = 608.33X − 1525, where Y represents peak intensity and X represents the concentration of the CA standard solution (μg/mL). The calibration range was 0.05–10 μg/mL, with a correlation coefficient of *R²* = 0.9976. Since authentic commercial standards for all hydroxycinnamic acid derivatives were unavailable, relative response factors (RRFs) of the detected compounds were experimentally determined using CA as the reference standard. The concentrations of 33 hydroxycinnamic acid-related compounds were subsequently estimated based on their peak responses and corresponding RRF values. The results were expressed as RRF-based estimated concentrations in the final extract (μg/mL).The detailed analytical validation parameters, including RRF values, RRF RSD, LOD, LOQ, recovery, and precision, are provided in **Supplementary Table S2**.

### 2.4 Analyses of phenotypic variation, correlations, and regional differences

Descriptive statistics, including the mean, standard deviation, and coefficient of variation, were calculated in R for the contents of the 33 hydroxycinnamic acid-related compounds. Pearson correlation analysis was performed to evaluate relationships among the compounds, and correlations were visualized using the R package PerformanceAnalytics (v2.0.4). To evaluate the effects of different agroecological regions on the accumulation of hydroxycinnamic acid-related compounds, a linear mixed-effects model was applied to the compound contents measured across three regions (WC, XZ, and JN). Region was treated as a fixed effect, whereas accession (genotype) was treated as a random effect to account for the repeated measurements of the same germplasm accessions across different environments. The model was fitted separately for each hydroxycinnamic acid-related compound, and the significance of regional effects, genotype variance, and residual variance were estimated. Differences were considered significant at *P* < 0.05 and highly significant at *P* < 0.01.

### 2.5 Comprehensive membership function value analysis

To comprehensively evaluate the overall capacity for hydroxycinnamic acid-related compounds accumulation among sweetpotato accessions, 152 accessions with phenotypic measurements available across the three agroecological regions were selected for MFVC analysis. Following previously reported methods, the content of each compound was standardized using a membership function, and the comprehensive membership function value (MFVC) was calculated (Zhang et al., 2025). Missing measurements were excluded without imputation. After membership function standardization, the mean membership function value of the available compounds was calculated for each accession and used as its MFVC. The number of valid compounds and trait coverage rate for each accession were recorded to evaluate the reliability of the comprehensive assessment. Based on their MFVCs, the accessions were classified into four categories: [0, 0.25), [0.25, 0.50), [0.50, 0.75), and [0.75, 1.00]. Elite accessions with a high overall capacity for hydroxycinnamic acid-related compounds accumulation were subsequently identified.

### 2.6 Genome-wide association study

GWAS was conducted using 4,599,509 high-quality SNPs obtained from previous whole-genome resequencing (Xiao et al., 2023). Association analysis was performed using the mixed linear model (MLM) implemented in the R package rMVP (v1.0.9). The kinship matrix (K matrix) and population structure matrix (Q matrix) were calculated using the integrated functions in rMVP. Population structure was estimated based on principal component analysis (PCA), and the first three principal components (nPC = 3) were included as fixed-effect covariates in the MLM model. The K matrix was used to account for genetic relatedness among accessions. The genome-wide significance threshold was determined using the Bonferroni correction based on the total number of SNP markers (4,599,509). The corrected threshold was calculated as *P* = 0.05/4,599,509, corresponding to approximately −log_10_(*P*) = 7.96. For convenience, this value was rounded to −log_10_(*P*) ≥ 8 as the genome-wide significance threshold for GWAS visualization and reporting. Based on the GWAS results from the three regions, SNP loci and candidate intervals (genomic regions defined by clusters of significant SNPs within stable or recurrent association regions) showing stable (consistently detected for the same traits across all three regional GWAS analyses) or recurrent (repeatedly identified in pairwise regional comparisons) associations were further identified.

### 2.7 Local linkage disequilibrium and genotypic effect analyses

Representative lead SNPs were selected for local linkage disequilibrium (LD) analysis. LD strength was measured using pairwise r² values between SNPs, and local LD heatmaps were generated. The germplasm accessions were grouped according to their lead-SNP genotypes, and the contents of the corresponding hydroxycinnamic acid-related compounds were compared among genotype groups. Differences between groups were assessed using an unpaired two-tailed Student’s t-test. Differences were considered significant at *P* < 0.05 and highly significant at *P* < 0.01. Statistical analyses and boxplot generation were performed using GraphPad Prism.

### 2.8 Candidate gene screening, functional annotation, and expression analysis

Candidate genes within the identified GWAS-associated intervals were identified based on their physical positions in the sweetpotato genome. For the stable association interval at 19.71–21.09 Mb on Iba_chr05a, all annotated genes within this interval were extracted. For the recurrent association signals on Iba_chr10a, annotated genes were extracted separately from the 24.88–25.10 Mb and 25.54–26.14 Mb intervals. The corresponding gene IDs, protein annotations, and ortholog information were provided in **Supplementary Data 1**. Using storage-root expression data from 88 sweetpotato accessions (Ding et al., 2017), the expression levels of selected genes located within the candidate intervals were analyzed. Among these accessions, 45 white-fleshed and 43 purple-fleshed sweetpotato accessions were used for comparison. The expression levels of candidate genes were normalized using FPKM values. Differences in gene expression between white- and purple-fleshed groups were evaluated using a two-tailed Welch’s unpaired t-test.

### 2.9 Expression analysis of candidate genes under abiotic stress conditions

To further evaluate the expression patterns of GWAS-identified candidate genes under abiotic stresses, transcriptome datasets of sweetpotato under drought, heat, and low-temperature stress conditions were analyzed. The drought- and heat-stress transcriptome datasets were obtained from Tang et al. (2023) (NCBI GEO accession: GSE216152). In this dataset, sweetpotato cultivar Xuzishu 8 was subjected to heat stress (42 °C, HT), drought stress (30% PEG6000 treatment, DR), and control conditions (CK). Three biological replicates were included for each treatment.

The low-temperature transcriptome dataset was previously generated by our laboratory from storage roots of Shangshu16 subjected to 6 °C storage treatment, while freshly harvested storage roots were used as controls. Three biological replicates were included for each treatment. RNA-seq data were processed by quality filtering, genome alignment, and transcript quantification. Gene expression levels were quantified using RSEM, and the complete gene expression matrix and annotation information of the low-temperature transcriptome dataset were provided as **Supplementary Data 2**.

Expression values of GWAS candidate genes under the corresponding control and stress treatments were extracted and standardized using row Z-score transformation for each gene. Heatmaps were generated using R to visualize the relative expression patterns of candidate genes under different abiotic stress conditions.

## 3 Results

### 3.1 Phenotypic variation in hydroxycinnamic acid-related compounds

A total of 33 hydroxycinnamic acid-related compounds were quantified in a sweetpotato germplasm panel comprising 229 accessions (**Supplementary Figure S1**). The number of accessions with valid measurements varied among compounds and environments because of differences in phenotypic data availability (**Supplementary Table S3**). Substantial variation in metabolite accumulation was observed among the evaluated accessions. Among the hydroxycinnamoylquinic acids, the mean contents of 1,5-diCQA, 1,5-diFQA, 3,4-diCQA, 3,5-diCQA, 3-CQA, 3-FQA, 4,5-diCQA, 4-FQA, 5-CQA, 5-FQA, and 5-pCoQA ranged from 0.14 to 0.64 μg/mL, with standard deviations of 0.03–0.89 μg/mL and coefficients of variation of 20.63%–139.44%. Ferulic acid-related derivatives, including 1-Caf-5-FerQA, 1-Fer-5-CafQA, 3-OMeRA, Fer-C1-GlcA, Fer-Gly, FA, FA-4-O-GlcA, FA-4-Sulf, isoFA, isoFA-3-O-GlcA, isoFA-3-Sulf, and isoFer-C1-GlcA, had mean contents of 0.18–0.64 μg/mL, standard deviations of 0.05–0.71 μg/mL, and coefficients of variation of 25.70%–110.53%. The coumaric acids m-CouA and p-CouA had relatively high mean contents, ranging from 1.03 to 1.04 μg/mL, with coefficients of variation of 99.14%–99.90%. Caffeic acid glycosides and other related compounds, including CA, CA-3-O-GlcA, CA-3-Sulf, CA-4-O-GlcA, CA-4-Sulf, Caf-C1-GlcA, RA, and SinA, had mean contents of 0.17–0.84 μg/mL and coefficients of variation of 16.12%–188.87%.Pearson correlation analysis showed that compounds within the same metabolic class generally exhibited coordinated accumulation patterns (**Figure 1**). Significant positive correlations were observed among the CQA derivatives 1,5-diCQA, 3,4-diCQA, and 3,5-diCQA, as well as between the FA-related derivatives 1-Caf-5-FerQA and 1-Fer-5-CafQA. Significant positive correlations were also detected between CQA derivatives and ferulic acid-related derivatives. Caffeic acid- and coumaric acid-related compounds showed varying degrees of positive correlation with CQA and ferulic acid derivatives. In contrast, Fer-Gly was significantly negatively correlated with several CQA derivatives, whereas RA and SinA showed weak correlations with most compounds.

**Figure 1:**
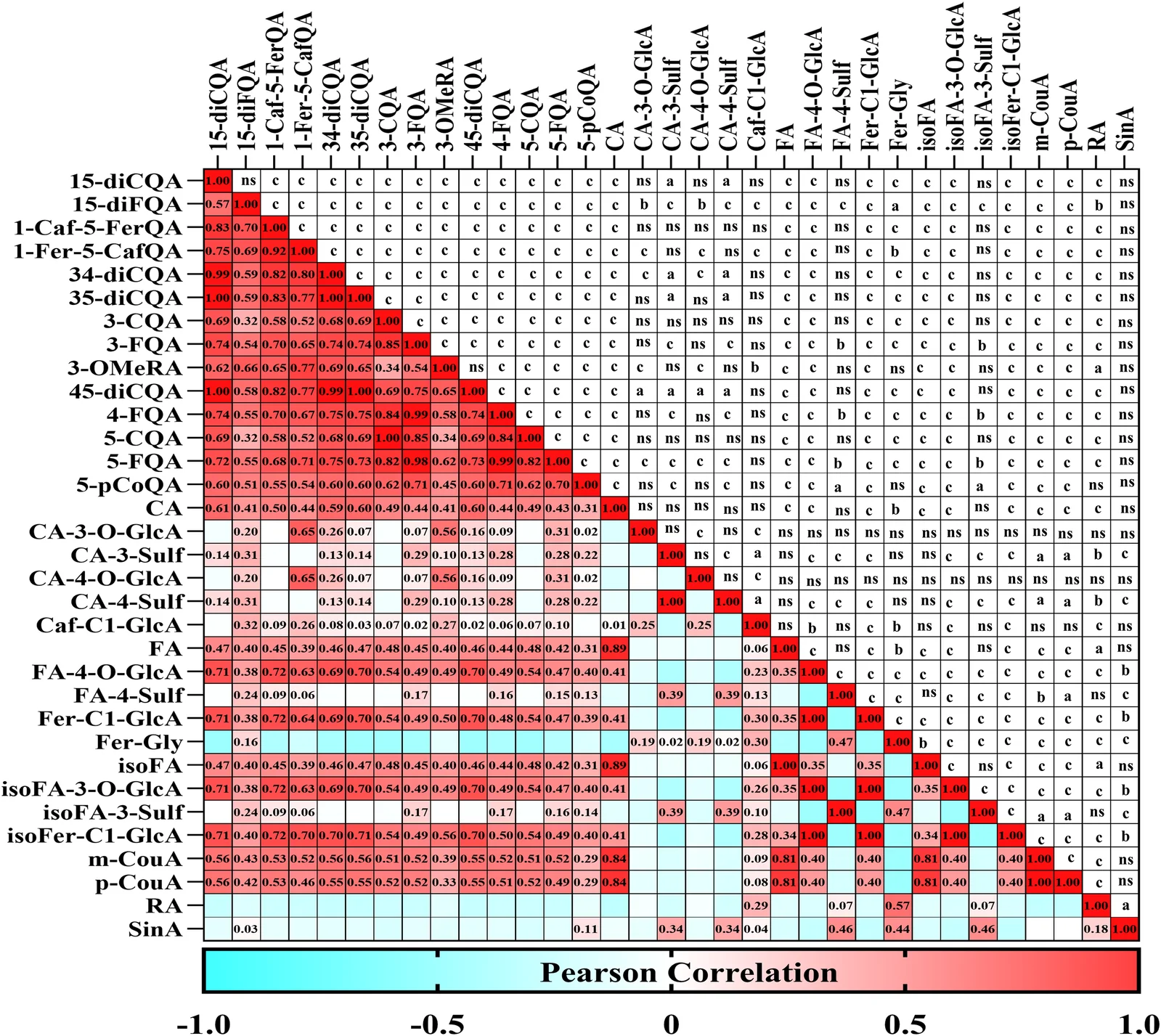
Pearson correlation analysis of the contents of 33 hydroxycinnamic acid-related compounds in sweetpotato storage roots. Values in the lower triangle represent Pearson correlation coefficients. Letters in the upper triangle indicate significance levels: a, P < 0.05; b, P < 0.01; c, P < 0.001; and ns, not significant. Red and cyan indicate positive and negative correlations, respectively, with darker colors representing stronger correlations.

### 3.2 Regional differences in hydroxycinnamic acid-related compounds

The regions showing the highest mean accumulation differed among compounds (**Supplementary Table S3**). Fourteen compounds had their highest mean contents in WC, 12 in XZ, and seven in JN. In contrast, 23 compounds had their lowest mean contents in JN, indicating generally lower accumulation of most compounds in this region. Specifically, p-CouA and m-CouA showed their highest mean contents in XZ and were 4.83- and 4.69-fold higher, respectively, than those observed in JN. CA showed its highest mean content in WC (1.084 μg/mL), approximately 3.29-fold that in JN.

To evaluate the effects of regional environments on hydroxycinnamic acid-related compound accumulation while accounting for genotype effects, a linear mixed-effects model was applied separately to the 33 hydroxycinnamic acid-related compounds measured in XZ, JN, and WC. Significant regional effects were detected for 31 compounds, whereas CA-3-O-GlcA and CA-4-O-GlcA showed no significant regional effects. The estimated variance components revealed differences in genotype variance and residual variance among compounds. Genotype variance ranged from 4.39 × 10^-^¹² to 3.09 × 10^-^¹, while residual variance ranged from 5.0 × 10^-4^ to 6.68 × 10^-^¹. Significant genotype variance was detected for most compounds, with relatively higher genotype variance observed for 3-CQA, 5-CQA, m-CouA, and p-CouA (**Supplementary Table S4**).

### 3.3 MFVC-based germplasm evaluation

Given that most hydroxycinnamic acid-related compounds differed significantly among the three regions, comprehensive membership function values (MFVCs) were calculated using accessions shared across all three regions to evaluate their overall accumulation capacity. The MFVC analysis included 152 sweetpotato germplasm accessions with measurements from XZ, JN, and WC. MFVCs ranged from 0.030 to 0.479, indicating substantial variation in the overall capacity for hydroxycinnamic acid-related compounds accumulation among the accessions (**Supplementary Table S5**). Based on the MFVC classification criteria, the accessions were divided into four intervals: [0, 0.25), [0.25, 0.5), [0.5, 0.75), and [0.75, 1]. Of the 152 accessions, 105 were assigned to the [0, 0.25) interval, accounting for 69.1%, whereas 47 were assigned to the [0.25, 0.5) interval, accounting for 30.9%. BZ13 had the highest MFVC, at 0.479. The 10 highest-ranking accessions were BZ13(WanYW-36-1), BZ31(Wanzishu56), BZ19(Ningzishu1), BZ20(Xuzishu2), BZ30(Mianyuzi11), BZ70(Lingzi), BZ17(Yanzishu2), BZ23(Luozishu1), BZ29(Mianzishu9), and BZ51(Guijingshu6). Their MFVCs were all within the [0.25, 0.5) interval, indicating relatively high overall capacities for hydroxycinnamic acid-related compounds accumulation within the tested population (**Table 1**). In contrast, BH60 had the lowest MFVC, at only 0.030, indicating a low overall accumulation capacity.

**Table 1:** Top 10 accessions ranked by MFVC.

Rank	Material ID	MFVC	Interval	Valid traits
1	BZ13(WanYW-36-1)	0.4794	[0.25, 0.5)	33
2	BZ31(Wanzishu56)	0.4518	[0.25, 0.5)	33
3	BZ19(Ningzishu1)	0.4456	[0.25, 0.5)	33
4	BZ20(Xuzishu2)	0.4450	[0.25, 0.5)	33
5	BZ30(Mianyuzi11)	0.4326	[0.25, 0.5)	33
6	BZ70(Lingzi)	0.4325	[0.25, 0.5)	33
7	BZ17(Yanzishu2)	0.4219	[0.25, 0.5)	32
8	BZ23(Luozishu1)	0.4074	[0.25, 0.5)	33
9	BZ29(Mianzishu9)	0.3993	[0.25, 0.5)	33
10	BZ51(Guijingshu6)	0.3972	[0.25, 0.5)	32

MFVC represents the mean membership function value calculated across valid hydroxycinnamic acid-related traits for each accession. A higher MFVC indicates a higher overall capacity for hydroxycinnamic acid-related compound accumulation. “Interval” indicates the MFVC range, and “Valid traits” indicates the number of traits included in the comprehensive evaluation.

### 3.4 Stable GWAS signals on Iba_chr05a

GWAS was performed for 33 hydroxycinnamic acid-related compound traits using a sweetpotato germplasm panel comprising 229 accessions, with the actual number of accessions varying among trait–environment combinations depending on the availability of valid phenotypic data. The QQ plots showed that the MLM adequately controlled the effects of population structure and kinship on the association analysis (**Supplementary Figure S2**). Using −log_10_(*P*) ≥ 8 as the significance threshold, 4,938 SNP markers significantly associated with the target traits were identified (**Supplementary Table S6**). Among all traits examined, the association signals for FA-4-O-GlcA and isoFer-C1-GlcA showed high stability across regions. Association signals for both traits were detected on Iba_chr05a in all three regions, and the significant signals showed substantial genomic overlap(**Figures 2A–C**). For FA-4-O-GlcA, the lead SNPs detected in the three regions were Iba_chr05a_20288229, Iba_chr05a_19790686, and Iba_chr05a_20357180, with corresponding *P* values of 3.68 × 10^-^¹¹, 4.24 × 10^-^¹³, and 1.31 × 10^-^¹^9^, respectively (**Table 2**). For isoFer-C1-GlcA, the lead SNPs detected in the three regions were located within the same association region on Iba_chr05a, with corresponding *P* values of 3.85 × 10^-^¹¹, 4.24 × 10^-^¹³, and 1.05 × 10^-^¹^9^, respectively (**Table 2**). Further integration of the genomic positions of significant SNPs showed that the major association signals for both traits were concentrated within the 19.71–21.09 Mb interval on Iba_chr05a, with 27 SNPs repeatedly detected across different regions (**Supplementary Table S7**).

**Figure 2:**
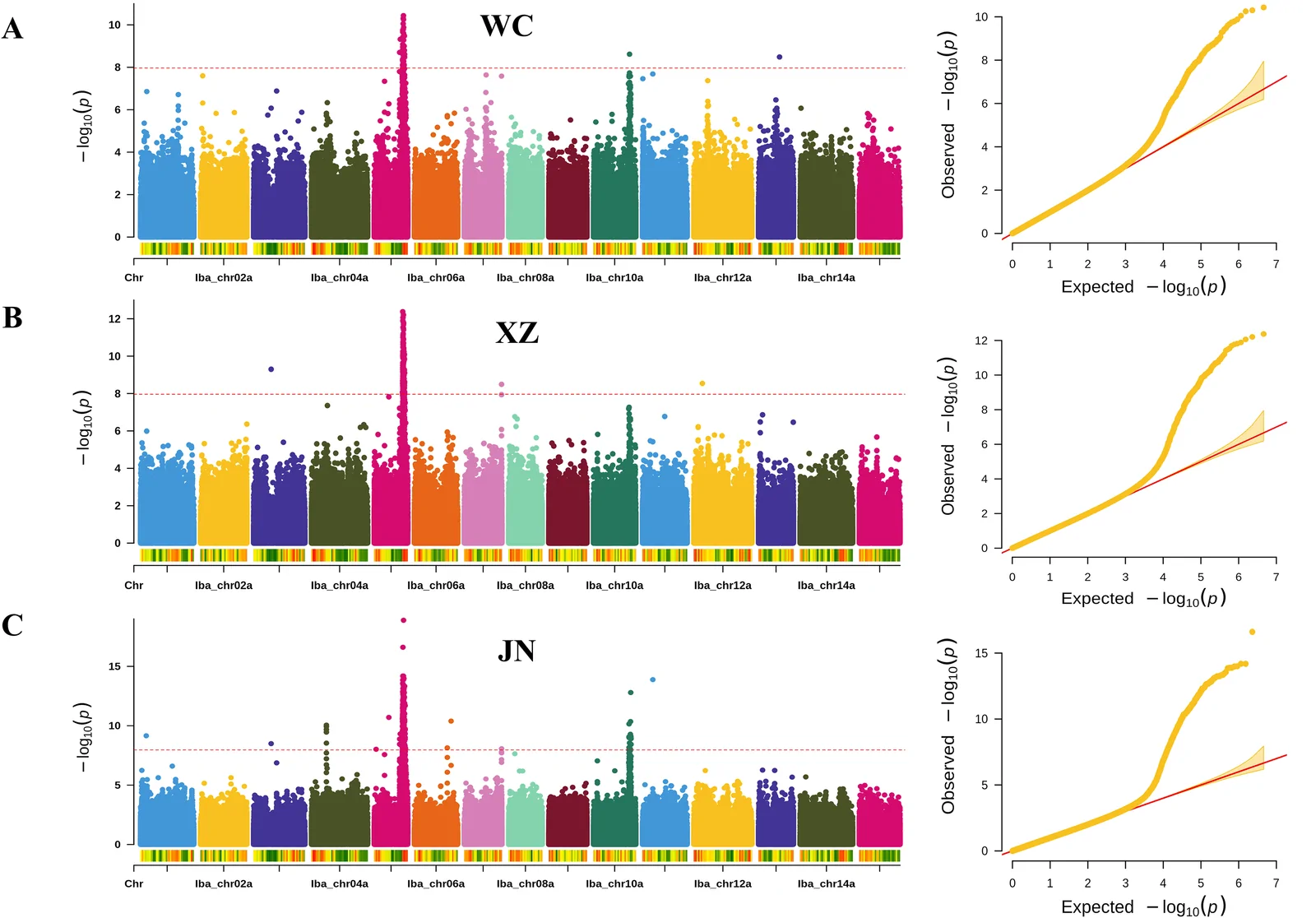
Genome-wide association analysis of compound FA-4-O-GlcA content in sweetpotato storage roots from three regions based on SNPs. **(A–C)** Hainan (WC), Xuzhou (XZ), and Shandong (JN), respectively. Left: Manhattan plots. The dashed horizontal lines represent the thresholds for significant associations. The x-axis displays the physical positions of all SNPs on the sweetpotato chromosomes, and the y-axis shows the −log_10_-transformed p-values for each association. Right: Q–Q plots of compound FA-4-O-GlcA content in the three regions.

**Table 2:** Stable GWAS signals on Iba_chr05a.

Parameter	FA-4-O-GlcA WC	FA-4-O-GlcA XZ	FA-4-O-GlcA JN	isoFer-C1-GlcA WC	isoFer-C1-GlcA XZ	isoFer-C1-GlcA JN
Region	WC	XZ	JN	WC	XZ	JN
Dataset	trait48	trait260	trait472	trait55	trait267	trait479
Total significant SNPs	53.00	141.00	318.00	55.00	141.00	356.00
SNPs on Iba_chr05a	51.00	138.00	288.00	53.00	138.00	319.00
Proportion	0.96	0.98	0.91	0.96	0.98	0.90
Lead SNP	Iba_chr05a_20288229	Iba_chr05a_19790686	Iba_chr05a_20357180	Iba_chr05a_20288229	Iba_chr05a_19790686	Iba_chr05a_20357180
Lead position (bp)	20,288,229.00	19,790,686.00	20,357,180.00	20,288,229.00	19,790,686.00	20,357,180.00
*P* value	3.68 × 10^-^¹¹	4.24 × 10^-^¹³	1.31 × 10^-^¹^9^	3.85 × 10^-^¹¹	4.24 × 10^-^¹³	1.05 × 10^-^¹^9^
−log_10_(*P*)	10.43	12.37	18.88	10.41	12.37	18.98
Stable interval	Iba_chr 05a: 19.71–21.09 Mb	Iba_chr 05a: 19.71–21.09 Mb	Iba_chr 05a: 19.71–21.09 Mb	Iba_chr 05a: 19.71–21.09 Mb	Iba_chr 05a: 19.71–21.09 Mb	Iba_chr 05a: 19.71–21.09 Mb

WC, Hainan; XZ, Xuzhou; JN, Shandong. Columns represent trait–region datasets. “Total significant SNPs” indicates the total number of significant SNPs detected for each trait in each region, whereas “SNPs on Iba_chr05a” indicates the number of significant SNPs located on Iba_chr05a. “Proportion” was calculated as the ratio of significant SNPs on Iba_chr05a to the total number of significant SNPs. “Lead SNP” represents the most significant SNP in each trait–region dataset. Trait48, trait260, and trait472 correspond to FA-4-O-GlcA content in WC, XZ, and JN environments, respectively, whereas trait55, trait267, and trait479 correspond to isoFer-C1-GlcA content in WC, XZ, and JN environments, respectively.

To further characterize linkage relationships among representative loci within the stable association interval on Iba_chr05a, local LD analyses were performed around the three lead SNPs. Local LD structures were observed around Iba_chr05a_19790686 (**Figure 3A**), Iba_chr05a_20288229 (**Figure 3C**), and Iba_chr05a_20357180 (**Figure 3E**), supporting these loci as important association signals within this interval. Iba_chr05a_20357180 showed the strongest association, and its surrounding region was therefore prioritized for subsequent candidate gene screening.

**Figure 3:**
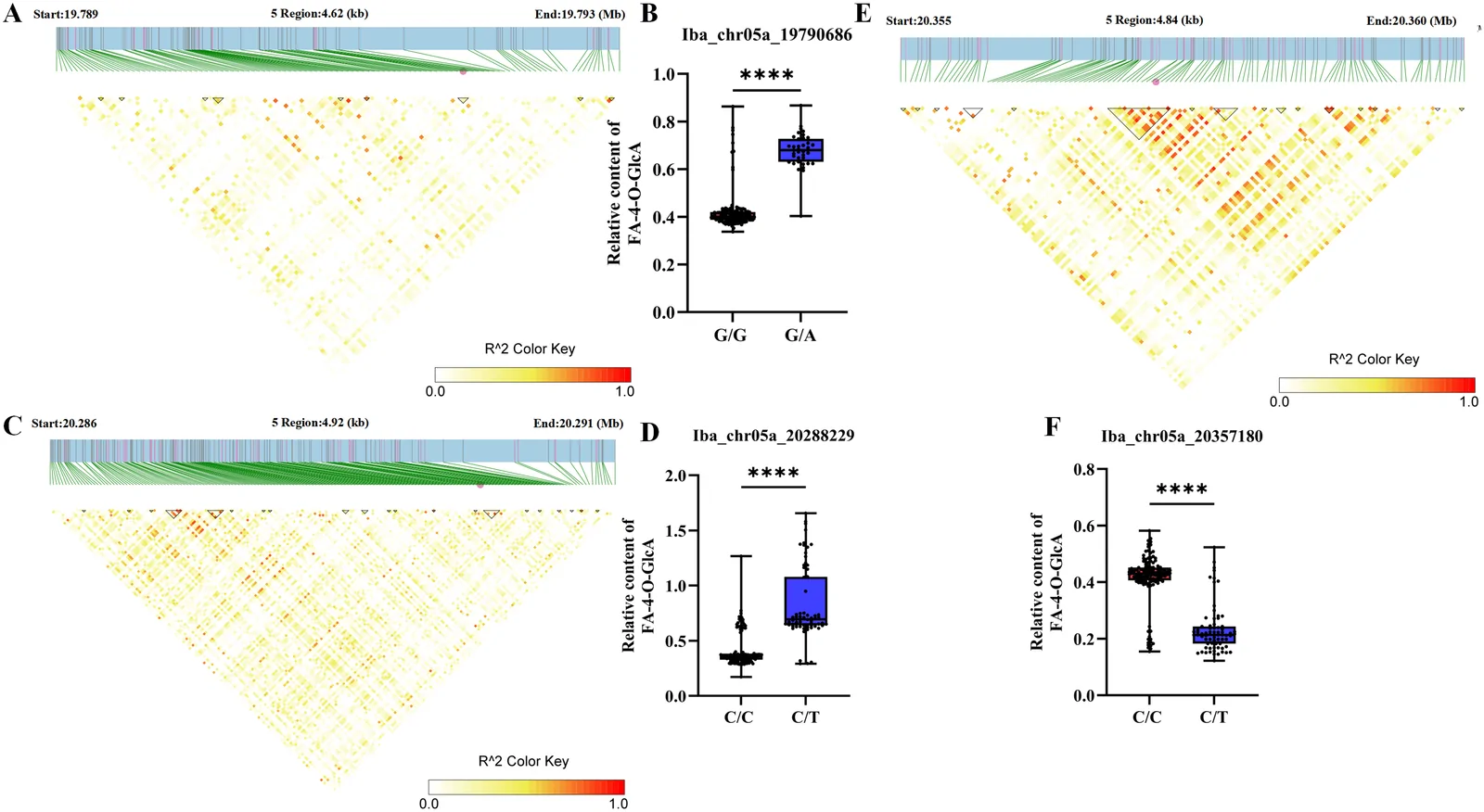
Local linkage disequilibrium and allelic effect analyses of significant SNPs associated with FA-4-O-GlcA content. **(A, C, E)** Local linkage disequilibrium (LD) heatmaps surrounding Iba_chr05a_19790686, Iba_chr05a_20288229, and Iba_chr05a_20357180, respectively. Colors ranging from light yellow to red indicate increasing LD strength (R²) between SNPs. **(B, D, F)** Differences in relative FA-4-O-GlcA content among accessions carrying different genotypes at the corresponding SNPs. Statistical differences between genotypes were determined using two-tailed Welch’s unpaired t-tests. ****P < 0.0001.

Genotypic effect analysis further showed highly significant differences in the relative content of FA-4-O-GlcA among the genotypes of all three lead SNPs. At Iba_chr05a_19790686, accessions with the G/A genotype had significantly higher relative FA-4-O-GlcA content than those with the G/G genotype (**Figure 3B**). At Iba_chr05a_20288229, accessions with the C/T genotype had significantly higher relative FA-4-O-GlcA content than those with the C/C genotype (**Figure 3D**). In contrast, at Iba_chr05a_20357180, accessions with the C/C genotype had significantly higher relative FA-4-O-GlcA content than those with the C/T genotype (**Figure 3F**). All three comparisons were highly significant. Collectively, the colocalized GWAS signals, local LD patterns, and genotypic effects support the 19.71–21.09 Mb interval on Iba_chr05a as an important candidate region associated with FA-4-O-GlcA and isoFer-C1-GlcA accumulation.

### 3.5 Recurrent GWAS signals on Iba_chr10a

Hydroxycinnamic acid traits repeatedly mapped to the same chromosomal region in two locations were further examined. Multiple traits were repeatedly mapped to Iba_chr10a in the XZ–WC or WC–JN regional comparisons (**Supplementary Table S8**). In XZ and WC, 1,5-diCQA, 1-Caf-5-FerQA, 1-Fer-5-CafQA, 3,4-diCQA, 3,5-diCQA, and 4,5-diCQA were repeatedly mapped to Iba_chr10a. The recurrent signals for 1,5-diCQA, 3,4-diCQA, 3,5-diCQA, and 4,5-diCQA were mainly concentrated around 24.88–25.10 Mb on Iba_chr10a, whereas those for 1-Caf-5-FerQA and 1-Fer-5-CafQA were primarily distributed around 25.54–26.14 Mb (**Supplementary Table S9**).

In WC and JN, 3-FQA, 4-FQA, 5-FQA, FA-4-O-GlcA, Fer-C1-GlcA, isoFA-3-O-GlcA, and isoFer-C1-GlcA were also repeatedly mapped to Iba_chr10a. The association signals for FA-4-O-GlcA, Fer-C1-GlcA, isoFA-3-O-GlcA, and isoFer-C1-GlcA were relatively consistent between the two regions. In WC, significant signals were mainly detected near Iba_chr10a_25079477, whereas an adjacent signal was detected near Iba_chr10a_25096457 in JN, together with a strong association peak at Iba_chr10a_25909723. Therefore, the 24.88–25.10 Mb interval on Iba_chr10a was prioritized as a candidate interval repeatedly detected in WC and JN, whereas the signal near 25.91 Mb was considered a potential secondary candidate locus.

To further evaluate the genetic effects of representative association loci on Iba_chr10a, local LD and genotypic effect analyses were performed around the major lead SNPs (**Supplementary Figure S3**). The LD heatmaps showed local LD structures around Iba_chr10a_24757103, Iba_chr10a_24875447, Iba_chr10a_25079477, Iba_chr10a_25087023, Iba_chr10a_25648613, Iba_chr10a_25661996, Iba_chr10a_25909723, and Iba_chr10a_26141562, supporting these loci as important association signals within the recurrent association regions on Iba_chr10a. Genotype-effect analysis revealed significant differences in metabolite contents at all eight lead SNPs (**Supplementary Figure S3**). For 1,5-diCQA, higher contents were observed in accessions carrying the G/A genotype at Iba_chr10a_24875447 and the C/T genotype at Iba_chr10a_25661996. For 3-FQA, higher contents were associated with the A/T genotype at Iba_chr10a_24757103 and the G/A genotype at Iba_chr10a_25087023. For isoFA-3-O-GlcA, the A/C genotype at Iba_chr10a_25079477 was associated with higher content. For 1-Caf-5-FerQA, higher contents were associated with the G/A genotype at Iba_chr10a_25648613 and the T/A genotype at Iba_chr10a_26141562. For Fer-C1-GlcA, the T/T genotype at Iba_chr10a_25909723 was associated with higher content.

### 3.6 Candidate gene annotation

Based on gene annotations within the stable association interval at 19.71–21.09 Mb on Iba_chr05a, a total of 191 genes were identified (**Supplementary Table S10**). Several of these genes were associated with phenylpropanoid metabolism, glycosylation, secondary metabolite transport, redox reactions, or transcriptional regulation. Iba_chr05a.1741 was annotated as a caffeoylshikimate esterase (CSE). The interval also contained UGT79B6, a cluster of UGT709C2-like glucosyltransferase genes, ABCG6, ABCB20, a GDSL esterase/lipase, the PMT11 methyltransferase, CYP90A1, and candidate transcription factors belonging to the DOF3.6, ZAT11, ABI4, and MYB (**Table 3**). Further annotation of the two candidate intervals on Iba_chr10a identified a total of 75 genes (**Supplementary Table S11**). The 24.88–25.10 Mb interval contained genes encoding a glyoxal/methylglyoxal oxidase, a probable glycosyltransferase, a DREB3-like transcription factor, an MKK6-like kinase, and a pectinesterase. The 25.54–26.14 Mb interval contained genes encoding a GATA transcription factor, a polyamine oxidase, a receptor-like protein kinase, a cytokinin dehydrogenase, and an AIM1-like protein associated with fatty acid β-oxidation (**Table 4**).

**Table 3:** Candidate genes in the Iba_chr05a region.

Gene ID	Position/Mb	Predicted annotation	Ortholog accession
Iba_chr05a.1692	19.776–19.779	GDSL esterase/lipase	Q8LB81
Iba_chr05a.1727	20.023–20.024	UDP-glycosyltransferase 79B6/UGT79B6	Q9FN26
Iba_chr05a.1728	20.028–20.029	UDP-glycosyltransferase 79B6/UGT79B6-2	Q9FN26
Iba_chr05a.1739	20.110–20.112	ABC transporter G family member 6/ABCG6	Q9FNB5
Iba_chr05a.1741	20.134–20.138	Caffeoylshikimate esterase/CSE	Q9C942
Iba_chr05a.1751	20.180–20.189	ABC transporter B family member 20/ABCB20	Q9M3B9
Iba_chr05a.1754	20.210–20.214	Zinc finger protein/ZAT11	Q9SLD4
Iba_chr05a.1767	20.317–20.318	Dof zinc finger protein/DOF3.6	Q9M2U1
Iba_chr05a.1803	20.542–20.546	Probable methyltransferase/PMT11	O22285
Iba_chr05a.1819	20.657–20.662	UGT709C2–1 like glucosyltransferase/UGT709C2-1	U3U992
Iba_chr05a.1820	20.667–20.670	UGT709C2–2 like glucosyltransferase/UGT709C2-2	U3U992
Iba_chr05a.1821	20.674–20.675	UGT709C2–3 like glucosyltransferase/UGT709C2-3	U3U992
Iba_chr05a.1822	20.676–20.679	UGT709C2–4 like glucosyltransferase/UGT709C2-4	U3U992
Iba_chr05a.1823	20.679–20.681	UGT709C2–5 like glucosyltransferase/UGT709C2-5	U3U992
Iba_chr05a.1854	20.903–20.909	Cytochrome P450 90A1/CYP90A1	Q42569
Iba_chr05a.1872	21.083–21.084	Ethylene-responsive transcription factor/ABI4	A0MES8
Iba_chr05a.1875	21.106–21.108	Putative MYB family transcription factor/MYB-like	Q700D9

Candidate genes were selected from the stable GWAS interval Iba_chr05a: 19.71–21.09 Mb and are ordered according to their physical positions. Functional annotations were assigned based on predicted protein functions and homologous gene information. Ortholog accession indicates the accession number of the homologous protein used for annotation. Gene ID represents the candidate gene identifier, and Position/Mb indicates the physical location of each gene within the candidate interval.

**Table 4:** Candidate genes in the Iba_chr10a intervals.

Gene ID	Position/Mb	Predicted annotation	Ortholog accession
Iba_chr10a.1085	24.881–24.882	Glyoxal/methylglyoxal oxidase/aldehyde oxidase GLOX1-like	I1S2N3
Iba_chr10a.1086	24.885–24.887	Glyoxal/methylglyoxal oxidase/aldehyde oxidase GLOX1-like	P0CS93
Iba_chr10a.1087	24.890–24.892	Glyoxal/methylglyoxal oxidase/aldehyde oxidase GLOX1-like	I1S2N3
Iba_chr10a.1088	24.893–24.895	Xyloglucan endotransglucosylase/hydrolase XTH26	Q9SVV2
Iba_chr10a.1089	24.904–24.910	Protein phosphatase 2C and kinase-domain protein/PP2C-kinase	Q9SL76
Iba_chr10a.1093	24.928–24.930	Probable glycosyltransferase/UGT	Q9FFN2
Iba_chr10a.1094	24.943–24.944	Ethylene-responsive transcription factor TINY-like/DREB3	Q9LYD3
Iba_chr10a.1106	25.091–25.094	Pectinesterase PME53/PME53	Q8VYZ3
Iba_chr10a.1108	25.102–25.105	MAP kinase kinase 6/MKK6	Q9FJV0
Iba_chr10a.1159	25.530–25.532	GATA transcription factor 22/GATA22	Q9SZI6
Iba_chr10a.1161	25.577–25.582	Beta-galactosidase-like/β-Gal-like	P48981
Iba_chr10a.1162	25.592–25.594	Polyamine oxidase 5/PAO5	Q9SU79
Iba_chr10a.1165	25.605–25.613	Receptor-like serine/threonine-protein kinase ALE2	Q8RWW0
Iba_chr10a.1171	25.668–25.670	Cytokinin dehydrogenase 3-like/CKX3	Q9LTS3
Iba_chr10a.1172	25.691–25.691	Cytokinin dehydrogenase 3-like/CKX3	Q9LTS3
Iba_chr10a.1176	25.729–25.733	Cytokinin dehydrogenase 3-like/CKX3	Q9LTS3
Iba_chr10a.1183	25.814–25.816	5-methyltetrahydropteroyltriglutamate-homocysteine methyltransferase/MetE-like	Q42662
Iba_chr10a.1187	25.874–25.875	AIM1-like peroxisomal fatty acid beta-oxidation protein/AIM1-like	Q9ZPI6
Iba_chr10a.1188	25.882–25.890	AIM1-like peroxisomal fatty acid beta-oxidation protein/AIM1-like	Q9ZPI6
Iba_chr10a.1192	25.947–25.951	AIM1-like peroxisomal fatty acid beta-oxidation protein/AIM1-like	Q9ZPI6
Iba_chr10a.1201	26.105–26.107	Receptor-like serine/threonine-protein kinase/RLK	Q6NKZ9

Candidate genes were selected from the stable GWAS interval Iba_chr10a and are ordered according to their physical positions. Predicted annotations were assigned based on homologous gene information. Ortholog accession indicates the accession number of the homologous protein used for annotation. Gene ID represents the candidate gene identifier, and Position/Mb indicates the physical location of each gene within the candidate interval.

### 3.7 Expression analysis of candidate genes

The expression levels of selected genes within the candidate intervals on Iba_chr05a and Iba_chr10a were further compared between white- and purple-fleshed sweetpotato accessions (**Figure 4**). Several candidate genes within the stable association interval on Iba_chr05a were differentially expressed between the two groups. *IbCSE*, *IbZAT11*, *IbUGT709C2-2*, and *IbMYB-like* showed higher relative expression levels in purple-fleshed accessions than in white-fleshed accessions, whereas *IbABCB20* and *IbPMT11* showed higher expression in white-fleshed accessions. *IbCYP90A1* showed no significant difference between the two groups. Within the recurrent association intervals on Iba_chr10a, *IbPP2C-kinase*, *IbUGT-like*, *IbDREB3*, *IbMKK6*, *IbGATA22*, *IbPAO5*, and *IbAIM1-like* showed higher relative expression levels in white-fleshed accessions than in purple-fleshed accessions.

**Figure 4:**
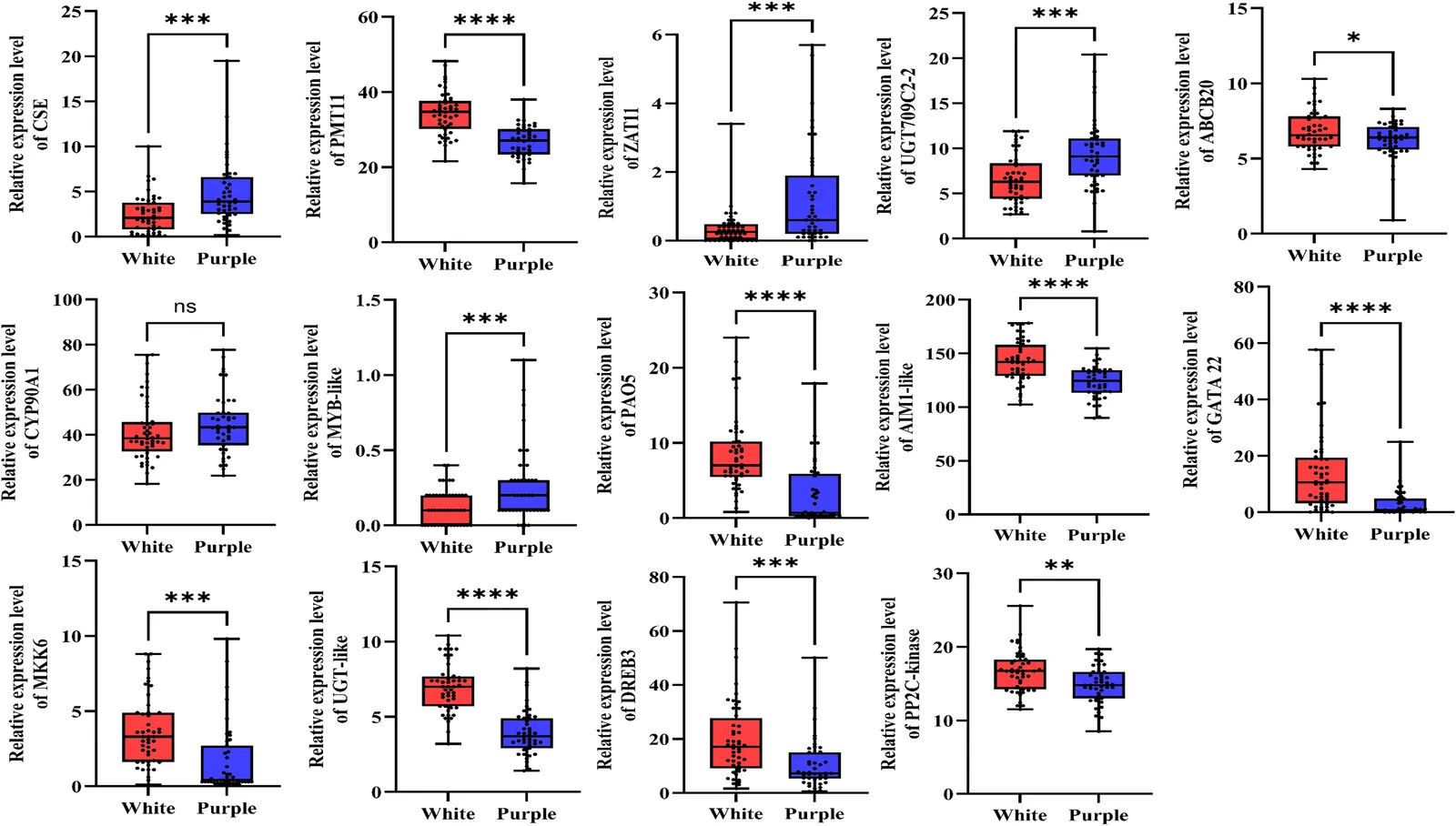
Comparison of candidate gene expression between white- and purple-fleshed sweetpotato accessions. Box plots show the relative expression levels of candidate genes, with red and blue representing white- and purple-fleshed accessions, respectively. Each point represents one accession. Statistical significance between the two groups was determined using two-tailed Welch’s unpaired t-tests. ns, not significant; *P < 0.05; **P < 0.01; ***P < 0.001; ****P < 0.0001.

The candidate genes also showed different expression patterns under abiotic stress conditions. *IbCSE*, *IbABCB20*, and *IbUGT709C2–2* exhibited relatively high expression levels under the 6 °C treatment, and *IbUGT709C2–2* was also upregulated under DR and HT treatments. *IbPMT11* and *IbZAT11* showed decreased expression under DR and HT treatments but increased expression at 6 °C compared with CK. *IbMYB-like* showed relatively high expression under DR and HT conditions but lower expression under the temperature treatments(**Figure 5A**). *IbMKK6*, *IbGATA22*, and *IbPAO5* were downregulated under both DR and HT treatments, with *IbMKK6* showing a more pronounced decrease under HT treatment. *IbUGT-like* and *IbDREB3* showed relatively high expression at CK and decreased at 6 °C, whereas *IbPP2C-kinase* and *IbAIM1-like* maintained relatively high expression levels under the temperature treatments(**Figure 5B**).

**Figure 5:**
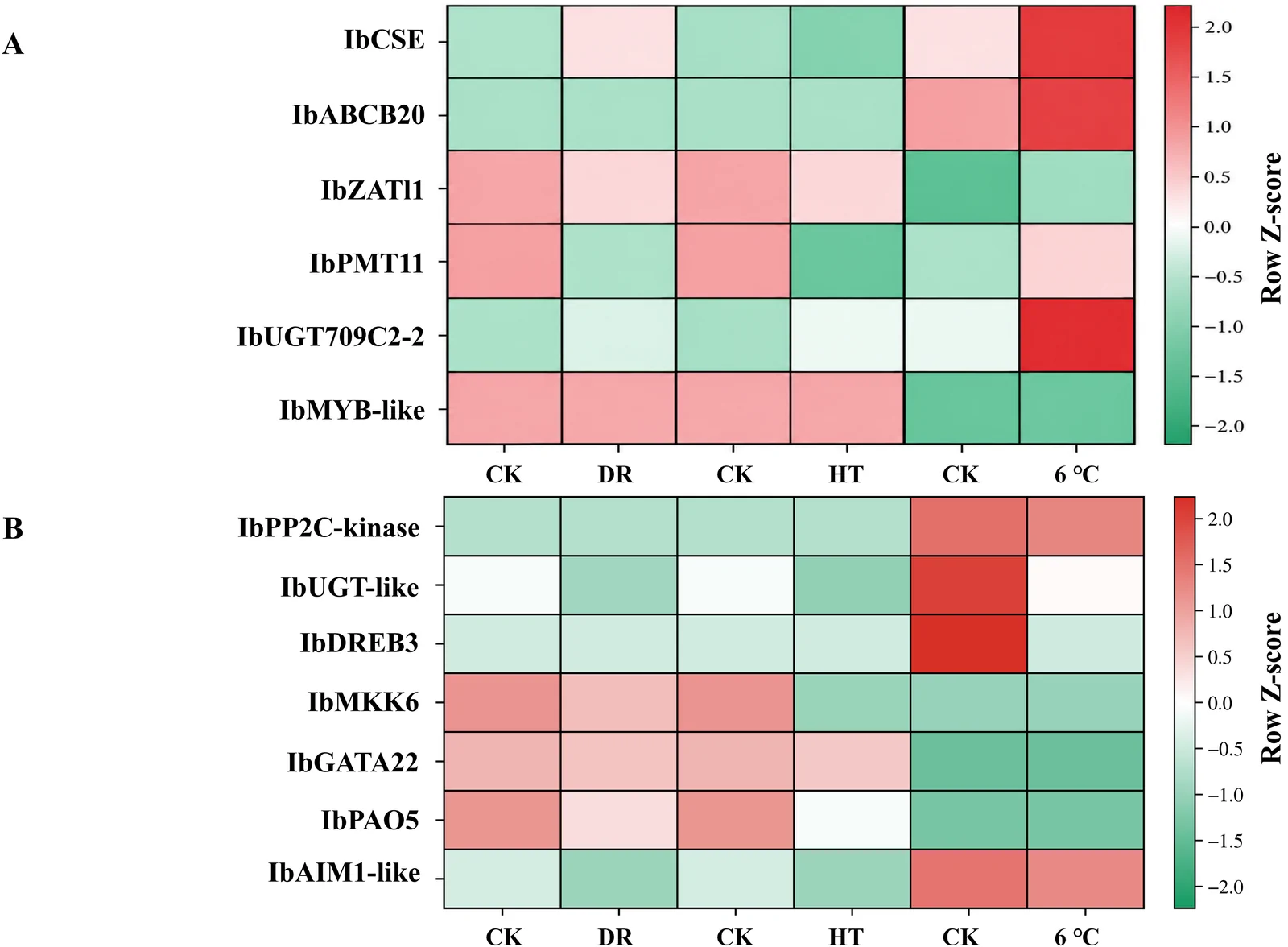
Expression profiles of candidate genes under abiotic stress conditions. **(A)** Expression patterns of candidate genes within the Iba_chr05a association interval. **(B)** Expression patterns of candidate genes within the recurrent Iba_chr10a association intervals. Gene expression was compared under drought (DR), high-temperature (HT), and 6°C treatments with their corresponding controls (CK). Expression values were standardized by row Z-score. Red indicates relatively high expression, whereas green indicates relatively low expression.

## 4 Discussion

Hydroxycinnamic acids are important bioactive compounds that influence the nutritional quality and functional value of crops (Sova and Saso, 2020). In sweetpotato storage roots, caffeoylquinic acids, coumaric acids, ferulic acid, and their derivatives are major phenolic acid metabolites closely associated with functional quality (Wan et al., 2024). Previous studies of phenolic acids in sweetpotato have focused primarily on compound identification, content comparisons, and antioxidant activity evaluation (Abugu et al., 2025). However, the genetic architecture and regulatory basis of these compounds in natural populations remain poorly understood, and relatively few associated loci and candidate genes have been identified. In this study, 229 sweetpotato germplasm accessions were subjected to phenotypic evaluation across three regions and LC–MS/MS quantification. The phenotypic variation and regional differences of 33 hydroxycinnamic acid-related compounds were systematically characterized, and stable loci, recurrently associated loci, and candidate genes were identified through GWAS. These findings provide further insight into the genetic basis of hydroxycinnamic acid accumulation in sweetpotato and an important reference for functional quality improvement, elite germplasm selection, and molecular breeding.

The 33 hydroxycinnamic acid-related compounds exhibited substantial variation across the population, with coefficients of variation ranging from 16.12% to 188.87%. This result indicates extensive diversity in phenolic acid accumulation among sweetpotato germplasm accessions and considerable potential for selecting elite accessions with high hydroxycinnamic acid contents. Correlation analysis revealed strong positive correlations among CQA, diCQA, FQA, and several ferulic acid derivatives. Because these compounds are closely associated with the phenylpropanoid pathway and share certain upstream precursors or modification processes (Yan et al., 2022a; Pappi et al., 2021), these positive correlations may reflect their coordinated accumulation in sweetpotato storage roots. Comparisons across regions showed that 31 compounds differed significantly among regions, suggesting that regional environments may influence their accumulation (Pazos et al., 2022). RA exhibited the strongest regional effect, consistent with previous findings that rosmarinic acid accumulation is sensitive to light, temperature, and water availability (Lee et al., 2023; Pistelli et al., 2019; Trócsányi et al., 2023). The MFVC-based comprehensive evaluation identified BZ13, BZ31, BZ19, BZ20, and BZ30 as accessions with high overall accumulation capacities, suggesting that they may represent valuable parental resources for subsequent functional quality breeding.

GWAS is an important approach for dissecting the genetic basis of complex quantitative traits (Guo et al., 2025b). In this study, an MLM was used to correct for population structure and kinship, thereby reducing the risk of false-positive associations (Dai et al., 2025). Compared with simpler models that do not fully account for relatedness, MLM provides a more conservative framework for association mapping and has been widely applied in sweetpotato GWAS studies (Bararyenya et al., 2020; Liu et al., 2022b). FA-4-O-GlcA and isoFer-C1-GlcA were consistently mapped to the 19.71–21.09 Mb interval on Iba_chr05a in all three regions. Multiple SNPs were repeatedly detected across regions and exhibited distinct LD patterns and genotypic effects, suggesting that this interval may represent a stable genetic region regulating the accumulation of ferulic acid-related derivatives. In contrast, associations on Iba_chr10a were predominantly detected in two regions and involved multiple traits, including CQA, FQA, and ferulic acid-related derivatives. Given that the effects of genetic loci may vary with environmental conditions (De Walsche et al., 2025), this region may exhibit relatively strong environmental dependence.

The stable association interval on Iba_chr05a contained *CSE*, *UGT79B6*, *UGT709C2-like*, *ABCG6*, *ABCB20*, and several transcription factors. *CSE* catalyzes the conversion of caffeoyl shikimate to caffeic acid and may regulate FA-4-O-GlcA and isoFer-C1-GlcA accumulation by affecting the formation of caffeic acid and downstream ferulic acid derivatives (Chen et al., 2025a). *UGT79B6* and *UGT709C2-like* may participate in the glycosylation of related compounds (Yonekura-Sakakibara et al., 2014; Miettinen et al., 2014), Members of the ABC transporter family have been shown to participate in the transport and storage of secondary metabolites (Yazaki, 2006). Although *ABCG6* and *ABCB20* are known to mediate the export of suberin barrier constituents and auxin efflux (Yadav et al., 2014; Zhang et al., 2018), respectively, their shared functional characteristics as ABC transporters suggest that they may also contribute to the transport and compartmentalized storage of modified metabolites, such as phenolic acids and flavonoids. In addition, MYB (Lin et al., 2021), DOF (Li et al., 2025c), ZAT (Zhao et al., 2026), and ABI4 (Guo et al., 2025a) transcription factors may contribute to this process by regulating phenylpropanoid metabolism or stress responses. The higher expression of CSE, UGT709C2-2, ZAT11, and MYB-like in purple-fleshed accessions further supports this possibility because anthocyanins and hydroxycinnamic acids share a common origin in the phenylpropanoid pathway (Vogt, 2010). Under abiotic stress conditions, *IbUGT709C2–2* was upregulated in response to drought, high temperature, and the 6 °C treatment. Plant UGTs are widely involved in abiotic stress responses, and individual UGT members show distinct expression patterns under drought, heat, and cold stress (Sun et al., 2022). *IbCSE* was also upregulated under drought and the 6 °C treatment. CSE functions in the phenylpropanoid pathway and contributes to monolignol and lignin biosynthesis (Wang et al., 2021b). Lignin deposition is closely associated with plant adaptation to drought, while enhanced phenylpropanoid and G-lignin biosynthesis has also been observed during low-temperature responses in several species (Choi et al., 2023). *IbZAT11* showed a different response pattern, with decreased expression under drought and high temperature but increased expression under the 6 °C treatment. A similar association between ZAT11 expression and drought response has been reported in oil palm, where ZAT11 was upregulated in the drought-sensitive genotype but downregulated in the more drought-tolerant genotype (Mejía-Alvarado et al., 2024). *IbMYB-like* maintained relatively high expression under drought and high-temperature conditions. In apple, *MdMYB88* and *MdMYB124* regulate phenylpropanoid and flavonoid metabolism and promote the accumulation of defense-related metabolites under drought stress (Geng et al., 2020).

Candidate genes identified within the recurrent association intervals on sweetpotato Iba_chr10a mainly included *PP2C-like*, *UGT-like*, *DREB3*, *MKK6*, *GATA22*, *PAO5*, and *AIM1-like*. Notably, these genes showed higher expression in white-fleshed accessions than in purple-fleshed accessions. This expression pattern may be related to differences in phenylpropanoid metabolism between contrasting flesh-color backgrounds (Wang et al., 2018). Purple-fleshed sweetpotato is generally characterized by enhanced flavonoid and anthocyanin biosynthesis (He et al., 2020), although this does not imply a simple competitive relationship with hydroxycinnamic acid metabolism. Anthocyanins and hydroxycinnamic acids share common precursors in the phenylpropanoid pathway, but their accumulation is controlled by complex regulatory networks rather than a simple metabolic trade-off (Vogt, 2010). Therefore, the contrasting flesh-color backgrounds provide a useful genetic context for evaluating the potential roles of GWAS-identified candidate genes in phenylpropanoid-related metabolism. Since phenylpropanoid-derived metabolites are important components involved in plant responses to environmental stresses, the expression patterns of these candidate genes under different abiotic stress conditions were further analyzed to explore their potential roles in stress responses (Dixon and Paiva, 1995). Functionally, these candidate genes are mainly associated with stress responses, signal transduction, ROS homeostasis, and peroxisome-related metabolism (Wang et al., 2025; Zhang and Zhang, 2022; Li et al., 2025b; Zhong et al., 2025). *IbMKK6*, *IbGATA22*, and *IbPAO5* were downregulated under both drought and high-temperature treatments, with *IbMKK6* showing a particularly pronounced decrease under high temperature. MAPK cascades are major components of environmental signal transduction in plants, and *GhMKK6* in cotton responds to drought, low-temperature, and high-temperature stresses (Zhang et al., 2016; Majeed et al., 2023). GATA transcription factors are also involved in abiotic stress responses, and *CmGATA22* in melon shows a clear transcriptional response to drought stress (Zheng et al., 2024). Polyamine oxidases participate in polyamine catabolism and ROS homeostasis, and *CsPAO5* in tea plants is responsive to drought stress (Li et al., 2020), in agreement with the drought-responsive expression of *IbPAO5* observed here. *IbUGT-like* was markedly downregulated under the 6 °C treatment, opposite to the response of *IbUGT709C2–2* within the Iba_chr05a interval, indicating gene-specific responses of different UGT members to low-temperature stress (Sun et al., 2022). *IbDREB3* was also downregulated under the 6 °C treatment, consistent with the established role of DREB transcription factors in temperature- and water-stress regulatory networks (Niu et al., 2020; Kidokoro et al., 2022). PP2C proteins are important regulators of ABA signaling and drought- and temperature-related responses, whereas AIM1 is involved in peroxisomal β-oxidation and H_2_O_2_-related metabolism (Wang et al., 2021a; van der Meer et al., 2020), providing a functional basis for the temperature-responsive expression of *IbPP2C-kinase* and *IbAIM1-like*. The significant SNPs and candidate genes identified in this study provide valuable genetic resources for understanding hydroxycinnamic acid accumulation and sweetpotato breeding. Future studies should validate these loci in independent breeding populations, perform haplotype analysis to identify favorable alleles, and develop functional markers for marker-assisted selection.

## Data Availability

The original contributions presented in the study are included in the article/**Supplementary Material**. Further inquiries can be directed to the corresponding author.
