# Supplementary material for: Genome-wide association analysis identifies candidate loci associated with hydroxycinnamic acid accumulation in sweetpotato
Source: Front Plant Sci. 2026 Oct 15;17:2001245. doi: 10.3389/fpls.2026.2001245 (PMC13716664; doi:10.3389/fpls.2026.2001245)
Supplement: Supplementary file 1 [file SupplementaryFile1.zip › Supplementary Material/Table 1.DOCX]

**Supplemental Figures**

**Figure S1.** Contents of 33 hydroxycinnamic acid-related compounds in the storage roots of sweetpotato germplasm accessions collected from three regions. JN, WC, and XZ represent Shandong, Hainan, and Xuzhou, respectively.

**Figure S2. Genome-wide association analyses of 33 hydroxycinnamic acid-related compounds traits across three environments.** trait27–trait59, trait239–trait271, and trait451–trait483 represent the 33 traits evaluated in WC, XZ, and JN, respectively. The traits correspond sequentially to 1,5-diCQA, 1,5-diFQA, 1-Caf-5-FerQA, 1-Fer-5-CafQA, 3,4-diCQA, 3,5-diCQA, 3-CQA, 3-FQA, 3-OMeRA, 4,5-diCQA, 4-FQA, 5-CQA, 5-FQA, 5-pCoQA, CA, CA-3-O-GlcA, CA-3-Sulf, CA-4-O-GlcA, CA-4-Sulf, Caf-C1-GlcA, FA, FA-4-O-GlcA, FA-4-Sulf, Fer-C1-GlcA, Fer-Gly, isoFA, isoFA-3-O-GlcA, isoFA-3-Sulf, isoFer-C1-GlcA, m-CouA, p-CouA, RA, and SinA. The upper panels show Q–Q plots, and the lower panels show Manhattan plots. The horizontal dashed line indicates the genome-wide significance threshold. The x-axis represents the physical positions of SNPs across the sweetpotato chromosomes, and the y-axis represents the −log₁₀(P) values of the association signals.

**Figure S3. Local linkage disequilibrium and genotypic effects of eight significant SNPs associated with hydroxycinnamic acid-related metabolite contents on chromosome 10a.** The heatmaps show pairwise linkage disequilibrium (LD) among SNPs surrounding each significant locus. Colors ranging from light yellow to red indicate increasing LD strength (R²). Box plots compare the relative metabolite contents among sweetpotato accessions carrying different genotypes at Iba_chr10a_24757103 (3-FQA), Iba_chr10a_24875447 (1,5-diCQA), Iba_chr10a_25079477 (isoFA-3-O-GlcA), Iba_chr10a_25087023 (3-FQA), Iba_chr10a_25648613 (1-Caf-5-FerQA), Iba_chr10a_25661996 (1,5-diCQA), Iba_chr10a_25909723 (Fer-C1-GlcA), and Iba_chr10a_26141562 (1-Caf-5-FerQA). Statistical differences between genotypes were determined using two-tailed Welch’s unpaired t-tests. ****, P < 0.0001.
